# Impact of integrative therapies on glycemic control in type 1 diabetes: a systematic review and global research landscape

**DOI:** 10.3389/fcdhc.2026.1739023

**Published:** 2026-04-20

**Authors:** Acharya Balkrishna, Jaya Upreti, Muskan Chauhan, Mayur Chauhan, Prashant Katiyar, Anurag Dabas, Vedpriya Arya

**Affiliations:** 1 Patanjali Herbal Research Division, Patanjali Research Foundation, Haridwar, Uttarakhand, India; 2 Department of Allied and Applied Sciences, University of Patanjali, Patanjali Yogpeeth, Haridwar, Uttarakhand, India

**Keywords:** glycemic control, integrative therapies, meta-analysis, systematic review, T1DM, type 1 diabetes

## Abstract

**Background:**

Type 1 diabetes mellitus (T1DM) requires lifelong insulin therapy due to autoimmune destruction of pancreatic β-cells, the difficulty of achieving ideal glycemic control despite advancements in conventional care has led to a rise interest in alternative and integrative medicine (CIM) practices like naturopathy, yoga, and Ayurveda.

**Objective:**

This systematic review and meta-analysis aimed to evaluate the effects of integrative therapies on glycemic control in individuals with T1DM and to map the global research landscape through bibliometric analysis.

**Methods:**

Following PRISMA, a thorough literature search was carried out in PubMed, Scopus, Embase, and Google Scholar. Included were studies evaluating CIM interventions for T1DM, such as yoga, naturopathy, and Ayurvedic treatments. Both qualitative and quantitative methods were used to synthesise the data, and bibliometric studies were conducted to evaluate country-level output, temporal trends, institutional contributions, and keyword network analysis.

**Results:**

Out 612 screened records, 12 studies met inclusion criteria for qualitative and quantitative analysis. Integrative therapies showed adjunctive benefits to insulin therapy, including improved glycemic control, insulin sensitivity, stress reduction and quality of life. After 2023, bibliometric study showed a growing research trend, In the context of citation impact, Italy and Vietnam are leading, whereas India leads in publications. Keyword network analysis revealed strong associations between integrative practices, particularly yoga and lifestyle modification and glycemic outcomes.

**Conclusion:**

Integrative therapies appear to be promising alternative in the management of T1DM, supporting glycemic control and holistic well-being. However, larger multicenter clinical trials are required to strengthen the evidence base and support their integration into standard diabetes care frameworks. Broader clinical integration, rigorous multicentric trials, and greater alignment with national AYUSH policies are recommended to optimize T1DM management in India and potentially enhance outcomes globally.

## 1 Introduction

**Figure 1:**
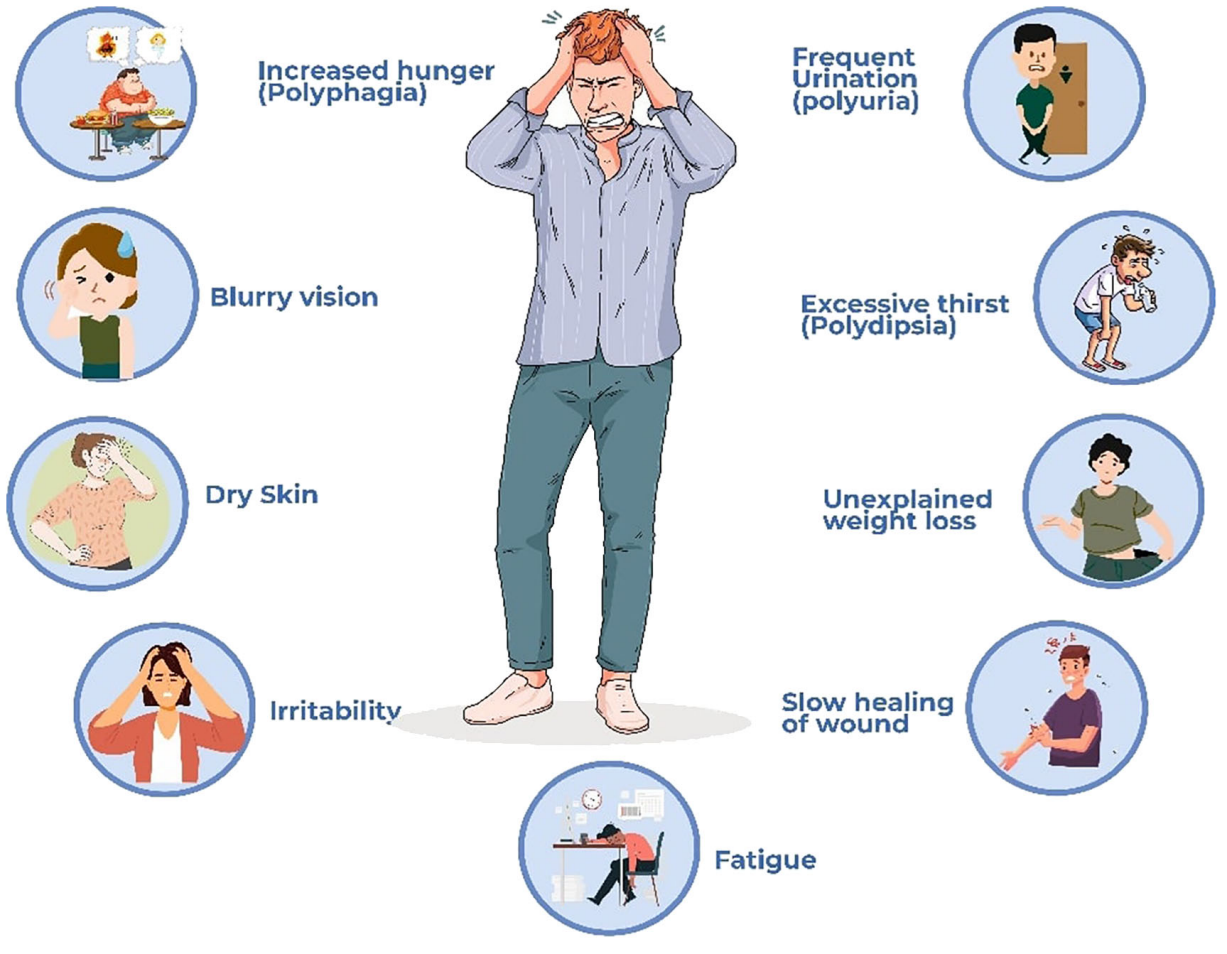
Different symptoms of type-1 diabetes mellitus.

Type 1 diabetes mellitus (T1DM) formerly called juvenile diabetes is a chronic lifestyle disorder characterized by the destruction of insulin-producing pancreatic β-cells, resulting in absolute insulin deficiency and dysregulated blood glucose levels (1) meanwhile, infrequent positive pancreatic autoantibody titers, leading to its classification as idiopathic (T1b) diabetes mellitus. There are three major types of diabetes: (a) Type 1, (b) Type 2 & (c) Gestational diabetes. The precise prevalence of diabetes types remains unclear due to limited distinction in prevalence surveys. However, the majority of diabetes cases are type 2, while type 1 cases are limited and must primarily involve children under age of 15 (2) and accounting for 7–10% of all diagnosed diabetes cases. It affects millions of individuals worldwide. However, the incidence of T1DM was notably higher in Scandinavia, Sardinia and Kuwait, and lower in Asia and Latin America (3). The India exhibits lower prevalence compared to Western nations but surpasses many other Asian countries, including China. Approximately one in five children worldwide diagnosed with T1DM is of Indian origin (4). Nonetheless, data are lacking for numerous countries. T1DM typically manifests in childhood or adolescence but can also occur in adults, often with slower onset and milder symptoms. Around 65,000 children receive a diagnosis of T1DM annually, with the incidence persistently rising by about 3% each year (5). This can be identified by metabolic dysregulations and characteristic clinical manifestations stemming from a continuing decline in insulin levels, usually occurring during an extended prodromal phase marked by the gradual depletion of pancreatic β-cells (6). Frequent urination, excessive thirst, or sudden weight loss, along with diabetic ketoacidosis (DKA), and low fasting C-peptide are common symptoms (**Figure 1**) (7). For centuries, Indian culture has embraced traditional healing practices such as Ayurveda, yoga, and naturopathy, providing holistic pathways to well-being (8). Dietary adjustments and herbal treatments are frequently employed to address a spectrum of health issues, including diabetes (9). There is a noticeable shift as individuals are now turning to integrated therapies to mitigate long-term complications and circumvent the high costs associated with modern medicine (10). Therefore, this systematic comparative review and meta-analysis aims to address this gap by examining the current evidence on the prevalence and efficacy of integrated therapies for managing T1DM in India. Through the analysis of these interventions, including their prevalence and impacts, the study endeavors to provide healthcare professionals with crucial insights, fostering a better comprehension of alternative strategies for diabetes management and potentially improving patient outcomes.

## 2 Search strategy and bibliometric framework

A bibliometric analysis was performed to identify and quantify literature reporting the prevalence of Type 1 Diabetes Mellitus (T1DM) in India, with emphasis on complementary and integrative medicine (CIM). Searches were conducted in PubMed, Scopus, Embase, and Google Scholar from inception to the most recent date using the Boolean query: (Diabetes 1 OR T1DM) AND (Prevalence of T1DM) AND (“Complementary and Integrative Medicine” OR CIM OR Yoga OR Naturopathy OR Ayurvedic therapies).

### 2.1 Study selection and eligibility criteria

**Figure 2:**
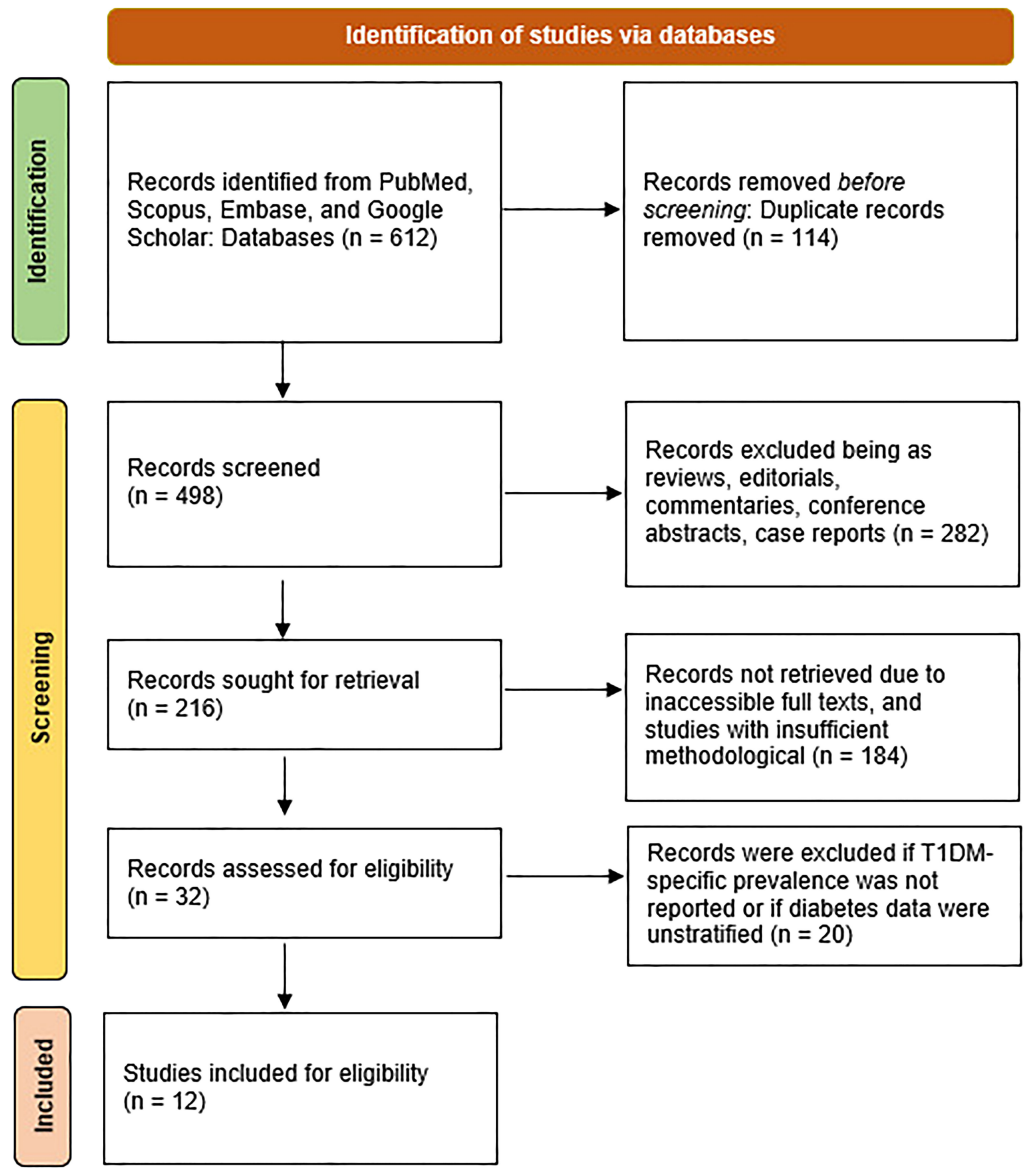
PRISMA flow-diagram for this study.

This systematic review followed the PRISMA 2020 guidelines to ensure transparency and reproducibility (11). Eligible studies reported the prevalence of T1DM and examined CIM approaches including Yoga, Naturopathy, or Ayurvedic therapies, and were peer-reviewed original research available in full text and indexed in PubMed, Scopus, Embase, or Google Scholar. Studies lacking T1DM-specific prevalence data, presenting unstratified mixed diabetes data, unrelated to CIM, or classified as reviews, editorials, commentaries, conference abstracts, case reports, duplicates, inaccessible full texts, or methodologically insufficient reports were excluded. A total of 612 records were identified from databases, and 114 duplicates were removed. Out of the remaining 498 records screened, 282 were excluded as reviews, editorials, commentaries, conference abstracts, or case reports. Among 216 records sought for retrieval, 184 were not retrieved due to inaccessible full texts or insufficient methodological quality. Consequently, 32 full-text articles were assessed for eligibility, of which 20 were excluded for lacking T1DM-specific prevalence data or presenting unstratified diabetes data. Finally, 12 studies met the inclusion criteria and were included in both qualitative and quantitative syntheses, as summarized in the PRISMA flow diagram (**Figure 2**).

### 2.2 Temporal trends (2020–2025)

**Figure 3:**
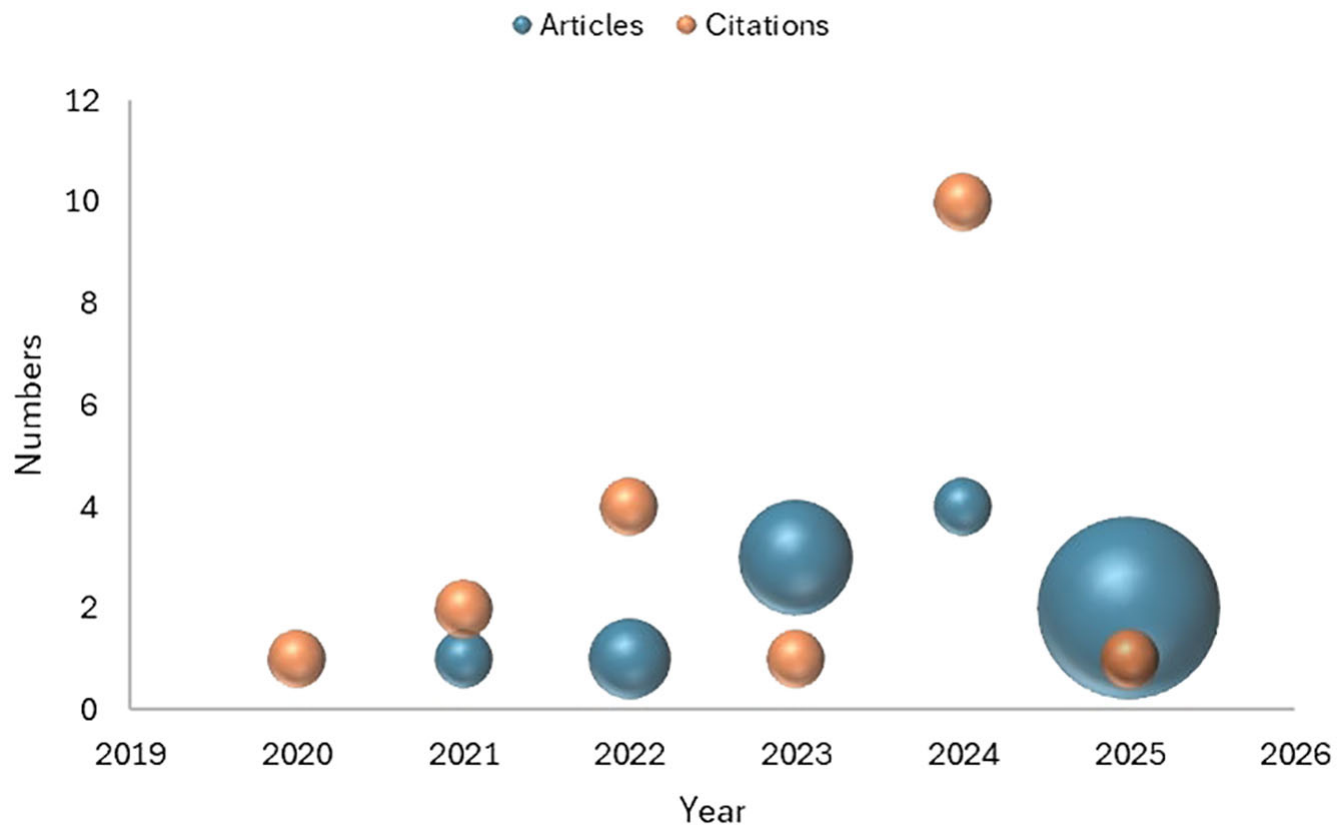
Temporal trends in research on integrative therapies for Type 1 Diabetes.

Research on integrative therapies for Type 1 Diabetes in India has steadily evolved, showing a gradual yet significant progression over the years. Between 2020 and 2022, scholarly publications were found to be limited, with only two articles published in 2020 (12), one in 2021 (13**),** and one in 2022 (14). However, citations during this period indicated growing interest, peaking in 2022 with four citations. A stronger growth phase followed in 2023 and 2024, during which a total of seven articles were published (15–21). Particularly, 2024 stood out as the most impactful year, with ten citations. By early 2025 (22, 23) two articles had already been published, though they had attracted only one citation, which may reflect a time lag in recognition or relatively lower influence compared to the 2024 peak (**Figure 3**). Overall, this trajectory suggests that integrative therapies for Type 1 Diabetes are a nascent but growing area of research in India, moving from exploratory work toward more impactful contributions, with 2024 marking a turning point for scholarly visibility.

### 2.3 Institutional contributions

**Figure 4:**
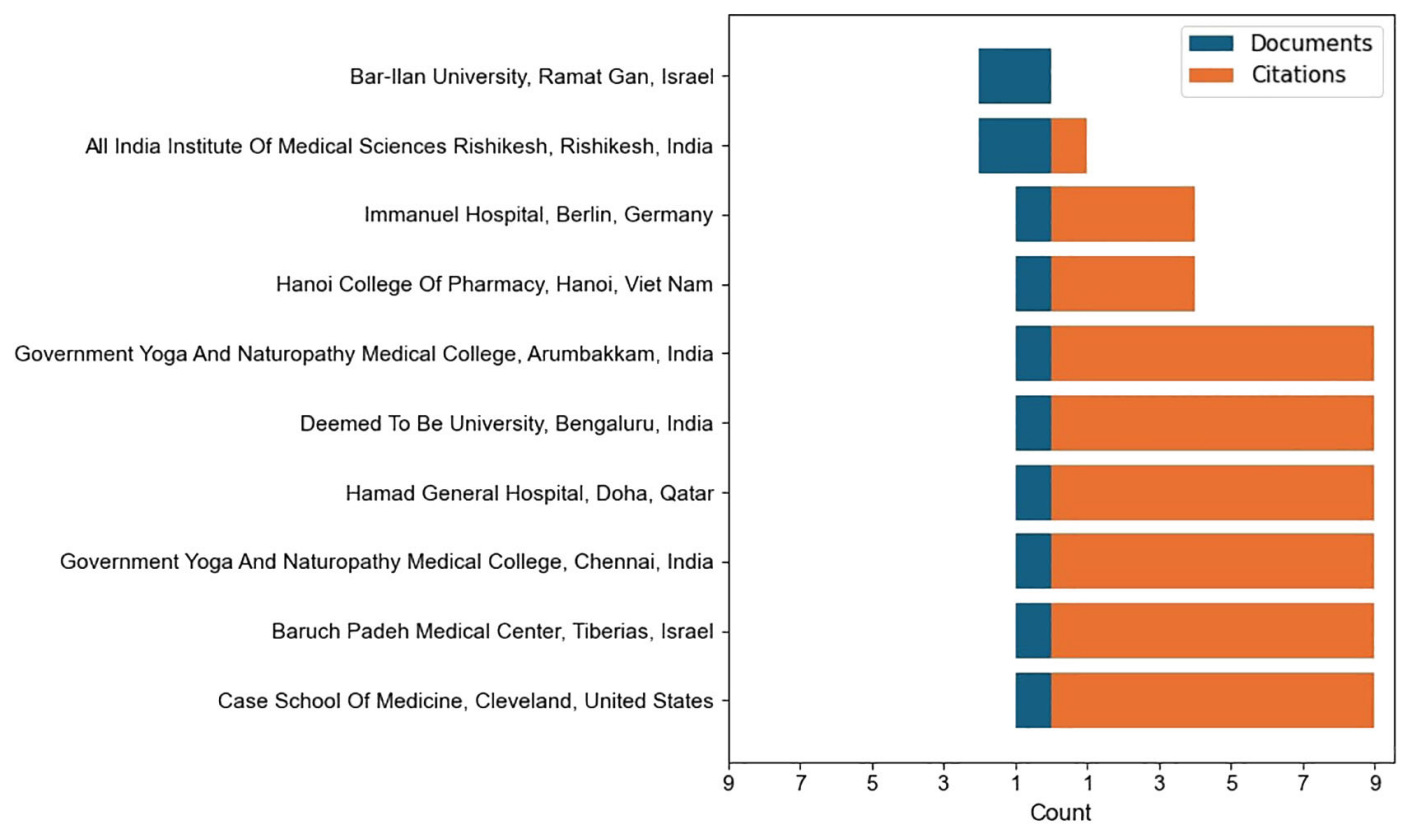
Institutional contributions with scholarly publications and highly cited studies.

Among Indian institutions, the All India Institute of Medical Sciences (AIIMS), Rishikesh, has been a key contributor with two documents and one citation, anchoring India’s formal academic involvement in this domain. Interestingly, smaller Indian institutions such as the Government Yoga and Naturopathy Medical Colleges in Chennai and Arumbakkam, as well as the S-VYASA University in Bengaluru, have produced single studies that achieved nine citations each highlighting the potential strength of traditional and naturopathic medicine research in shaping global discourse. Internationally, institutions such as Bar-Ilan University in Israel produced two documents but did not attract citations while Baruch Padeh Medical Center in Israel, the Case School of Medicine in the United States, and Hamad General Hospital in Qatar recorded highly cited single publications, each receiving nine citations. Similarly, Hanoi College of Pharmacy in Vietnam and Immanuel Hospital in Germany contributed impactful work with four citations each (**Figure 4**). This pattern represented that while AIIMS Rishikesh provides credibility as a recognized Indian institute, smaller specialized institutions in both India and abroad are producing high-quality, high-impact contributions to the field.

### 2.4 Country-level analysis

**Figure 5:**
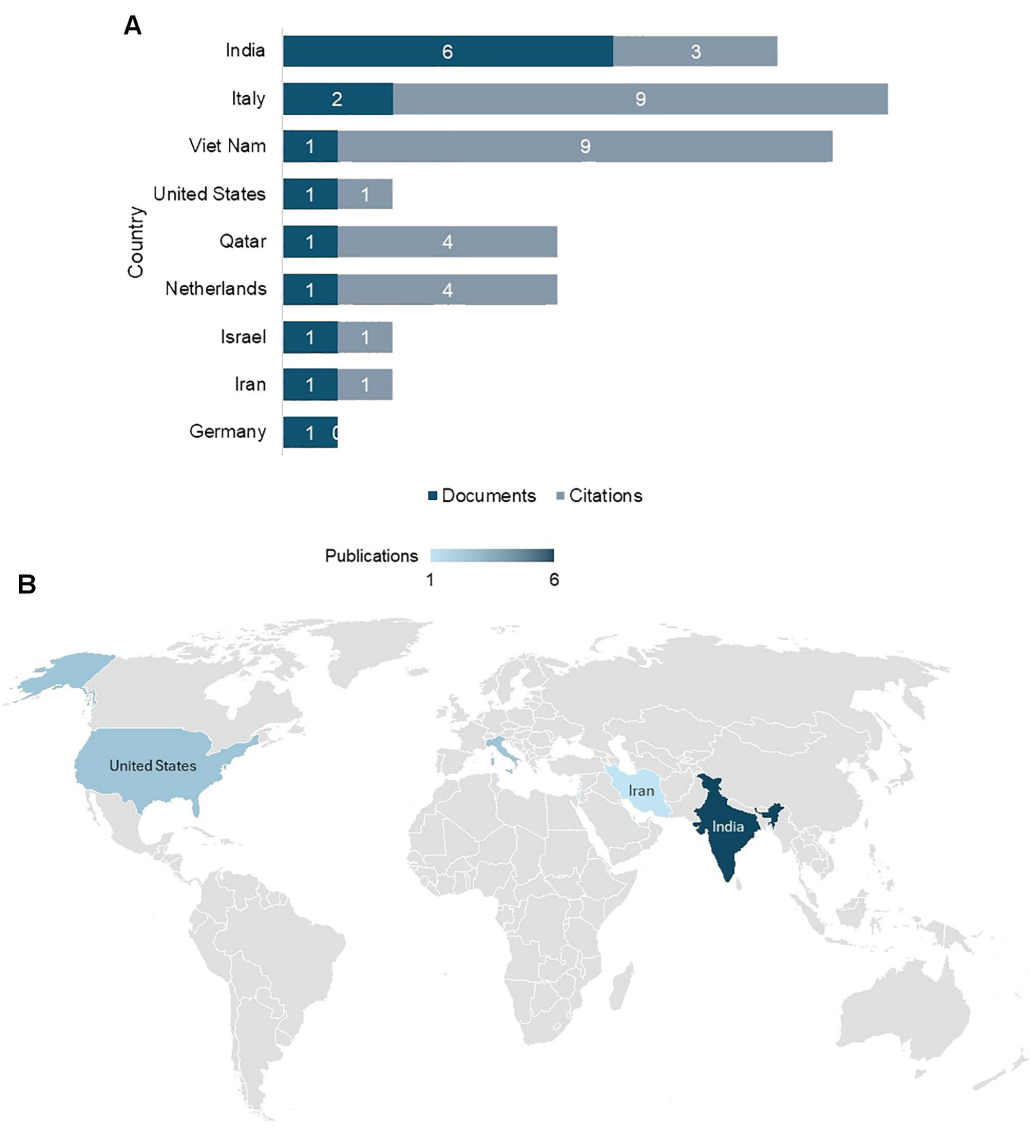
**(A)** Country-level contributions with impactful studies and citations. **(B)** Country-level scholarly publications on integrative therapies for Type 1 Diabetes.

At the country level, India leads in publication volume with six documents, though its citation count remains modest at three, reflecting a strong focus on building foundational literature rather than generating high-impact outputs. In contrast, Italy and Vietnam stand out as high-impact contributors, with Italy producing two documents that gained nine citations and Vietnam achieving the same impact with a single document (**Figure 5A**). The Netherlands and Qatar also showed relatively strong influence with four citations each, despite limited document counts. Conversely, research from Germany, Israel, and the United States produced lower impact with zero to one citation despite their established biomedical research infrastructure (**Figure 5B**). These findings revealed a clear pattern: India dominates in quantity, but smaller contributors like Italy and Vietnam are setting benchmarks in terms of quality and global recognition.

## 3 Keyword network analysis

**Figure 6:**
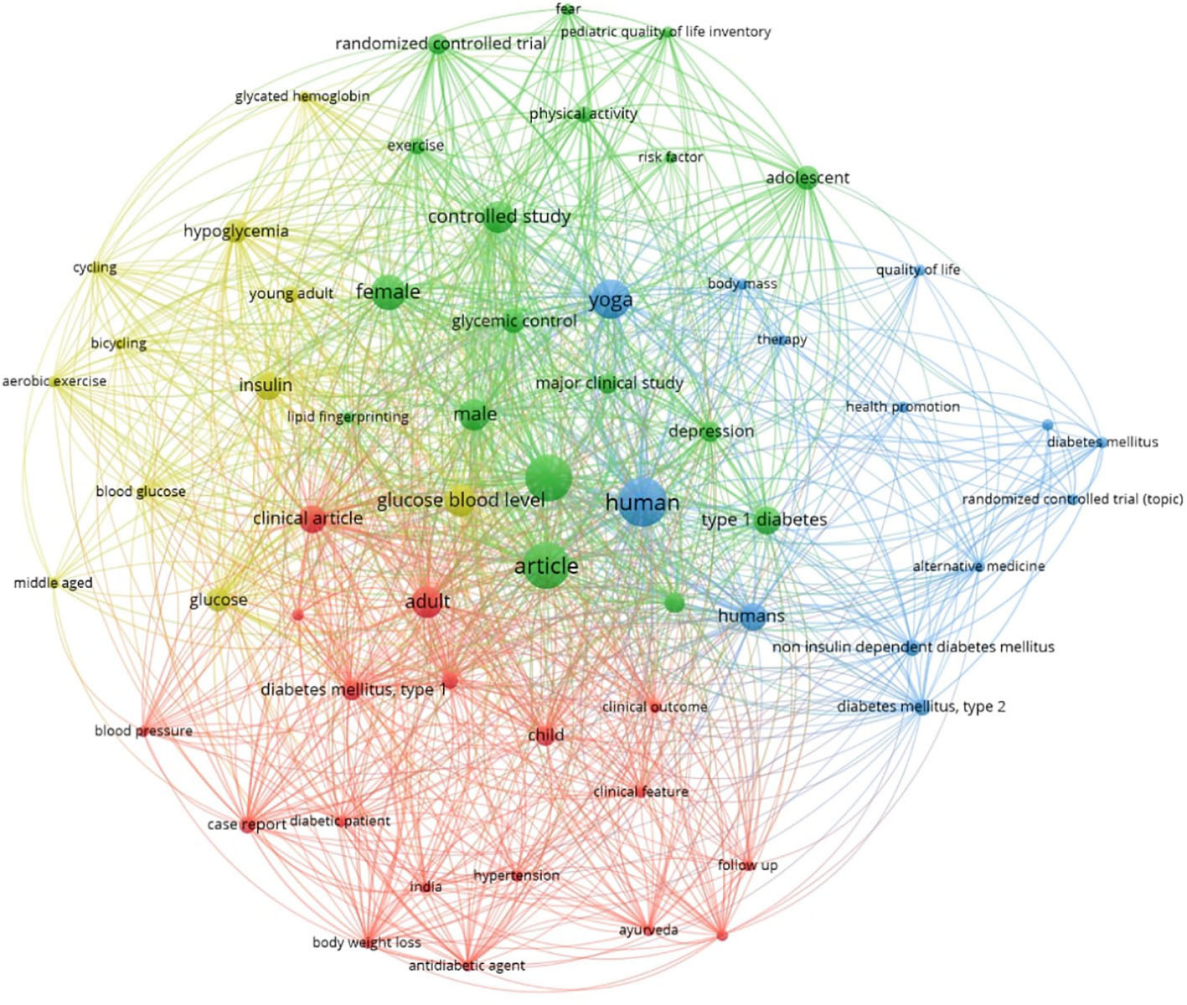
Keyword co-occurrence network of integrative therapy research in Type 1 Diabetes.

Keyword co-occurrence analysis with VOSviewer provided deeper insights into the conceptual structure of this emerging field. Out of 277 total keywords, 60 met the minimum threshold of two occurrences, forming distinct thematic clusters. The green cluster emphasized clinical trials, glycemic control, insulin use, and hypoglycemia management, suggesting that integrative therapies are often examined in conjunction with biomedical markers and conventional treatment. The blue cluster, prominently featuring yoga, health promotion, and alternative medicine, highlighted the centrality of lifestyle and mind–body interventions. The red cluster, centered on Type 1 diabetes mellitus, Ayurveda, antihyperglycemic agents, and India, represented region-specific approaches where traditional medicine intersects with chronic disease management. Finally, the yellow cluster links exercise, aerobic activity, and glucose monitoring, reinforcing the importance of physical activity as a complementary strategy (**Figure 6**). The prominence of terms such as human, clinical article, and clinical study points to an empirical, patient-centered evidence base. Collectively, the keyword network demonstrated that research in India and globally is positioning integrative therapies not as alternatives, but as complementary interventions aligned with clinical outcomes and patient quality of life.

India is emerging as a key contributor to research on integrative therapies for Type 1 Diabetes (24), though its global impact remains modest compared to countries like Italy and Vietnam, which have produced fewer but more influential studies. High-quality contributions are coming not only from major institutes but also from smaller institutions, highlighted the diversity of research strength. International collaborations with Vietnam, Italy, and Qatar have proven particularly impactful, highlighting the value of global partnerships. While India currently leads in research volume, the next step is to enhance influence through large-scale multi-center clinical trials, systematic reviews, and meta-analyses that can consolidate the evidence base. Alignment with AYUSH policies and integration into global diabetes care frameworks will be crucial. The keyword analysis indicates that yoga, Ayurveda, and lifestyle modifications are increasingly tied to biomedical markers, reflecting a shift toward holistic, evidence-based management. If India leverages its traditional strengths alongside rigorous clinical validation and international collaboration, it can position itself as a global leader in integrative diabetes care.

## 4 Causal factors of T1DM

Early-onset T1DM which accounts for 5%–10% of all diabetes cases, often leads to long-term complications (25). Despite extensive research, no single cause has been definitively identified, although many research is underway and showing many possible factors responsible for T1DM. A recent study has linked T1DM to higher intake of foods containing nitrosamines, nitrates or nitrites, as well as elevated nitrate levels in water samples from families with affected children (26). The likelihood of developing T1DM over one’s lifetime fluctuates based on familial influences, children face elevated risks of developing the condition if their mother (3%), father (5%), or sibling (8%) have been diagnosed with the disease. Genetic predisposition, particularly the HLA-DR3-DQ2 and HLA-DR4-DQ8 haplotypes in combination with environmental factors, is also believed to contribute to the development of T1DM (27, 28). Diabetic individuals face an increased risk of both macrovascular (coronary artery disease, peripheral vascular disease and stroke) and microvascular (diabetic nephropathy, retinopathy, and neuropathy) complications, collectively referred to as diabetic vascular complications. Globally, diabetes stands as the primary contributor to coronary artery disease, limb amputations, end-stage renal disease (ESRD), and blindness in comparison to those without diabetes (29) (**Table 1**). The onset of clinical symptoms indicates the advanced stage of β-cell destruction (30), Oxidative stress is pivotal in diabetes progression, causing cellular damage preceding complications. It diminishes insulin sensitivity, deteriorates pancreatic insulin-producing cells, and exacerbates mitochondrial DNA damage, impairing pancreatic β-cell function, largely through reactive oxygen species (ROS) generation by free fatty acids (35). Prolonged endoplasmic reticulum stress hampers insulin synthesis, leading to pancreatic β-cell apoptosis and reduced insulin production. Factors such as rapid growth, obesity, puberty, sedentary lifestyle, trauma, infections, and high glucose intake significantly contribute to the onset of T1DM (36, 37). The weight of the mother during pregnancy had the biggest impact on the chances of the child developing T1DM later on. For every unit increase in the mother’s BMI, the risk of T1DM in the child went up by 27%. Additionally, previous research has suggested a higher likelihood of developing T1DM in children born via Caesarean section and to older mothers compared to those born naturally and to younger mothers (38).

**Table 1:** Different causal factors and their mechanism of action of T1DM.

Causal factors	Mechanism of action	Reference
Genetic Predisposition	Presence of specific human leukocyte antigen (HLA) genotypes, such as HLA-DR3-DQ2 and HLA-DR4-DQ8, which increase susceptibility to autoimmune destruction of pancreatic β-cell	(30)
Autoimmune Response	Stimulation of T lymphocytes by environmental factors like viral infections causes pancreatic β-cell destruction via cytotoxicity and cytokine release. Meanwhile, disruption of self-antigen tolerance occurs due to the imbalance between regulatory and effector T-cells, ultimately causing autoimmune destruction of pancreatic β-cells.	(31)
Dietary factors	Dietary factors, such as early exposure to cow’s milk or gluten, may influence immune responses and contribute to autoimmunity through molecular mimicry or by microbiota dysbiosis (disruption of gut barrier function which altered microbiota composition)	(32)
Environmental & Psychological Factors	Environmental pollutants like bisphenol A (BPA) and phthalates disrupt immune function and contribute to pancreatic inflammation. Variations in T1D incidence across different regions imply that climate, sunlight exposure, and gut microbial diversity may play influential roles. Moreover, psychological stressors have the potential to worsen immune dysregulation and inflammation, potentially influencing the development of T1DM.	(33).
Other Factors	Oxidative stress: Increased production of reactive oxygen species (ROS) contributes to pancreatic β-cells damage and apoptosis.	(34)

### 4.1 Lifestyle and environmental risk factors for T1DM

Emerging evidence suggests that lifestyle elements may play a role in triggering T1DM (39). The current literature shows how lifestyle choices influence the risk of developing T1DM. Factors like diet, exercise, exposure to environmental pollutants, and psychological stress have their potential effect on T1DM vulnerability (40). A study conducted in Poland suggests that environmental factors play a crucial role in initiating autoimmune responses in the pancreas’s beta cells, particularly among children (41). Factors such as excessive screen time and poor dietary habits as well as diet high in carbohydrates have been linked to insulin resistance and obesity, which exacerbate metabolic issues in individuals with T1DM. Poor diet, consuming high levels of trans fats and additives like monosodium glutamate found in fast food, leading to cardiovascular and metabolic issues such as inflammation and insulin resistance (42), while smokers typically have higher HbA1c levels and shorter partial remission periods (43). Lifestyle choices among adolescents amplify the risk of depression, potentially triggering metabolic problems and associated complications (44). Conversely, inadequate glycemic control and resulting complications can further deteriorate their quality of life (45, 46). Although stressful life events, like family losses, sadness are linked to a higher risk of T1DM development in children, especially aged 5–9 years (47). Findings reveal that individuals with low genetic susceptibility as well as those consumed more sweets, candy and sugary beverages are prone to have T1DM (48, 49).

## 5 Lifestyle modification for improvement in T1DM

Lifestyle modification plays a pivotal role in enhancing diabetes management. It encompasses dietary adjustments, regular exercise, Yoga & Pranayama, stress reduction techniques, and adequate sleep (50). By adopting these ones can reduce the risk of complications and improve overall quality of life.

### 5.1 Physical activity

Physical activity improves the well-being of individuals with T1DM, engaging in physical activity can lower oxidative stress and enhance endothelial function, potentially improving insulin sensitivity which also enhances fitness, decreases insulin needs, improves lipid profiles, and enhances vascular endothelial function in T1DM patients (51). Research indicates that exercise enhances blood glucose control, lowers daily insulin needs, and mitigates the risk of diabetes-related complications in individuals with T1DM. Aerobic exercises specifically reduce insulin requirements for glycemic control and manage fasting blood-glucose level (52).

### 5.2 Yoga & pranayama

Yoga demonstrates its advantages in enhancing nerve conduction and cognitive functions in diabetic patients, thereby aiding in the effective management of diabetic complications (53). Yoga has been observed to enhance insulin sensitivity in target tissues, thereby reducing insulin resistance and boosting the peripheral utilization of glucose. Additionally, it can rejuvenate or regenerate pancreatic β-cells and heighten the sensitivity of these cells to glucose signals.

### 5.3 Diet

Increased carbohydrate and sugar intake elevate the risk for islet autoimmunity (IA) and subsequent T1DM (15). High-fat diets, regardless of fatty acid composition, decrease insulin sensitivity in humans. Saturated fat seems to be more harmful for inducing fat-related insulin insensitivity (54). Adolescents with T1DM are advised emphasizing the consumption of fruits, vegetables, whole grains and low-fat foods for a healthful diet (55, 56).

### 5.4 Sleep

Melatonin, produced by the pineal gland, plays a crucial role in regulating the sleep-wake cycle. Inadequate sleep duration and low sleep quality are linked to insulin resistance and impaired glucose metabolism (57, 58). Evening exercise, such as Tai Chi, can enhance sleep onset and quality while reducing daytime sleepiness (59). Acupuncture, known for its pain-relieving effects through neurotransmitter release and dopamine facilitation makes sleep better (60). Mind-body practices like guided imagery and meditation and alternative methods like aromatherapy, warm baths, and relaxing music hold potential for improving sleep patterns. Meanwhile, Yoga reduces anxiety and promotes better sleep (61).

## 6 Complementary and integrative medicine for diabetes care

Complementary and Integrative Medicine (CIM) signifies a pivotal transformation within healthcare, wherein traditional medical methodologies are harmoniously merged with empirically supported complementary therapies, thereby providing comprehensive and patient-centric healthcare approach (62). CIM therapies offer a diverse range of approaches for managing T1DM, emphasizing holistic well-being and addressing its root causes, while not replacing conventional treatments, although they can complement medical interventions to enhance health outcomes and improve quality of life (63). These therapies have gained popularity in recent years, especially in India, where research on integrative antidiabetic therapies has been on extensive mode (64). The Medical Expenditure Panel Surveys (MEPS) revealed that individuals diagnosed with diabetes were 1.6 times inclined to utilize CIM compared to those not diagnosed with diabetes (65). CIM emphasizes a holistic approach to health, integrating mind, body, and spirit through practices such as herbal medicine, acupuncture, yoga, meditation, reflexology and dietary supplements (66). CIM provides personalized treatment plans, designed to meet each patient’s unique needs and preferences by expanding the understanding of effective interventions and their therapeutic mechanisms (67). Here are some important CIM therapies that widely used for the treatment and management of T1DM (68).

### 6.1 Yoga

Yoga, a 3,000-year-old ancient practice originating from India, has gained global recognition for its profound effects on physical, mental, and emotional well-being as holistic health care, classified by the National Institutes of Health as Complementary Integrative Medicine (CIM) (69). Beyond its reputation for enhancing flexibility and reducing stress, yoga has emerged as a promising adjunct therapy for managing chronic conditions like Type 1 Diabetes (T1DM) (70). The integration of yoga into T1D management holds significant promise in improving overall health outcomes and quality of life for individuals with this condition. Different yoga therapies including Asanas (physical poses), Pranayama (breathing exercises) and meditation can positively affect various aspects of managing T1D (15). Yoga offers a holistic approach to managing Type 1 Diabetes by stabilizing blood sugar, reducing stress, regulating insulin, and supporting emotional well-being also triggers a physiological sequence of event in the body that reduces the stress response (**Table 2**) (15, 71). By understanding both the physiological and psychological impacts of yoga, now a day’s peoples use it’s as a potential complementary management for T1DM (72).

**Table 2:** Various yoga poses and pranayama that aid in the management of Diabetes (15).

ASANA	Method	POSE
Surya Namaskar	To perform Surya Namaskar: Start in Tadasana (Mountain Pose), then move through a sequence of poses including Forward Fold, Plank, Cobra or Upward Dog, and Downward Dog, coordinating each movement with inhalation or exhalation. Repeat the sequence 10 Times.	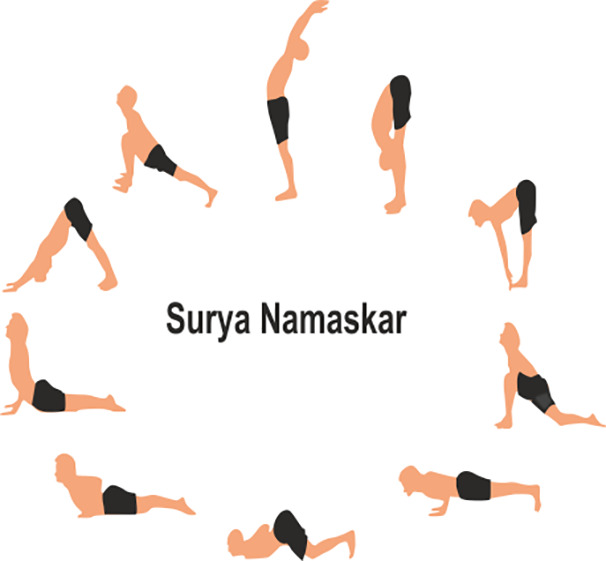
Shavasana	It’s also known as Corpse Pose. First, lie flat on your back than relaxed your arms and legs, focusing on deep breathing and releasing tension. It’s often practiced at the end of a yoga session at least 15 min.	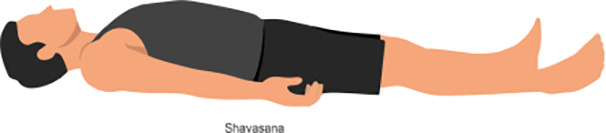
Vakrasana	Vakrasana, or Spinal Twist Pose, is a seated yoga posture where practitioner firstly twist their spine, bringing one arm across the opposite knee while placing the other hand behind for support. (Do it for 2-5min)	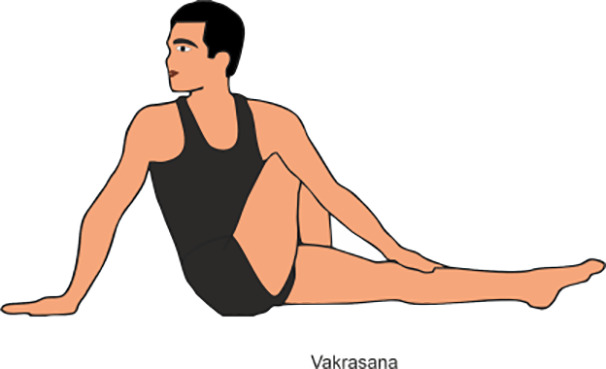
Ardha matsyendrasana	Ardha Matsyendrasana, or Half lord of the fishes Pose, is a seated yoga twist where practitioner sit with one leg bent and the other crossed over, twisting torso and placing the opposite elbow on the outer knee for support. (Do it for 2 min)	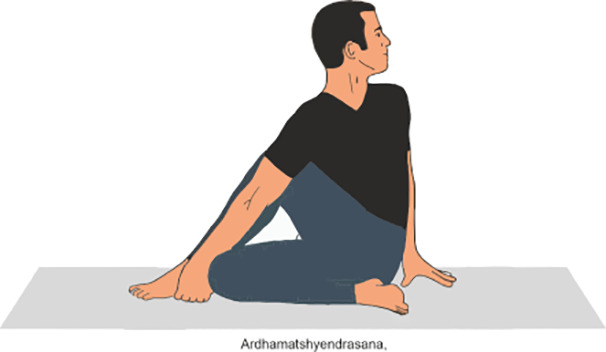
Gomukhasan	Gomukhasana, or Cow Face Pose, is a seated yoga posture where practitioner stack one knee over the other, bringing the feet to opposite sides of the hips, and then interlock the arms behind the back, one from below and the other from above.(Do it for 2 min)	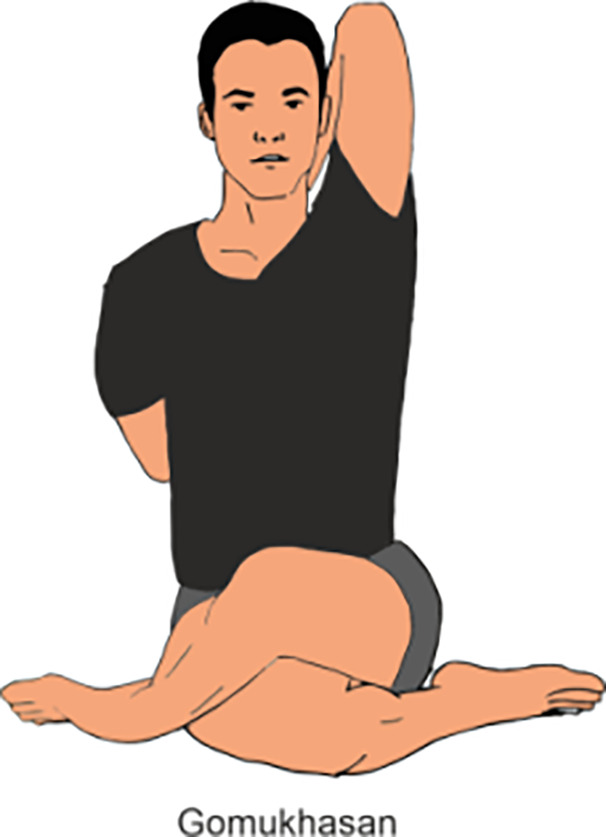
Mandookasan	Mandukasana, or Frog Pose, is a yoga posture where practitioner squat with knees wide apart, heels touching, and lean forward with hands on the floor, bringing the chest toward the ground. (Do it for 5 min)	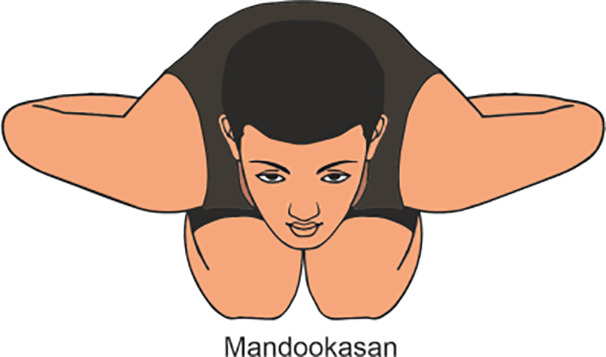
Yogmudrasan	Yogamudrasana, or Yoga Seal Pose, is a seated yoga posture where practitioner sit with legs crossed, then reach both arms behind the back, clasping the hands together. Bending forward, then lower the forehead toward the ground. (Do it for 10-15 min)	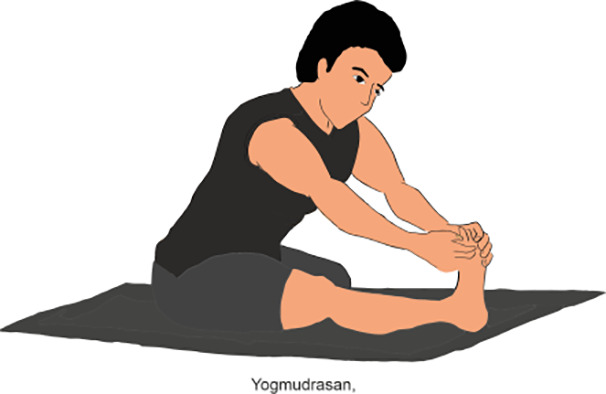
Vajrasana	Vajrasana, or Thunderbolt Pose, is a kneeling yoga posture where practitioner sits on heels, keeping the spine straight and hands resting on the thighs stay on this posture near ¼ minute to one minute.	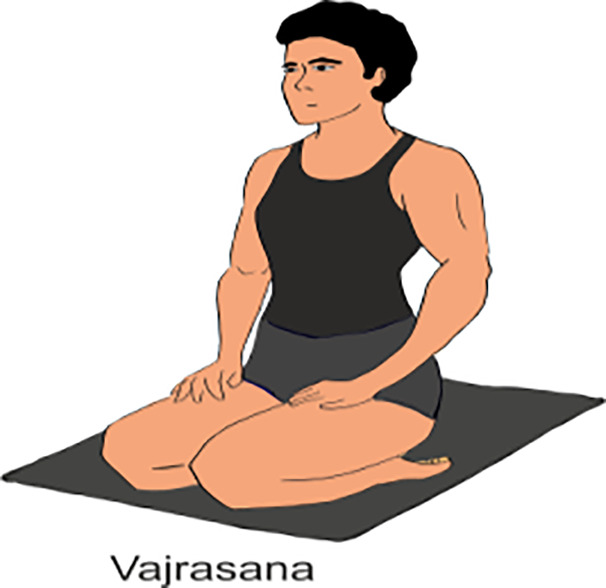
PRANAYAMA	METHOD	POSE
Bhastrika	Sit comfortably, Firstly, practitioner inhale deeply and forcefully through nose, then exhale rapidly with equal force.(Do it 3-5 mins per day)	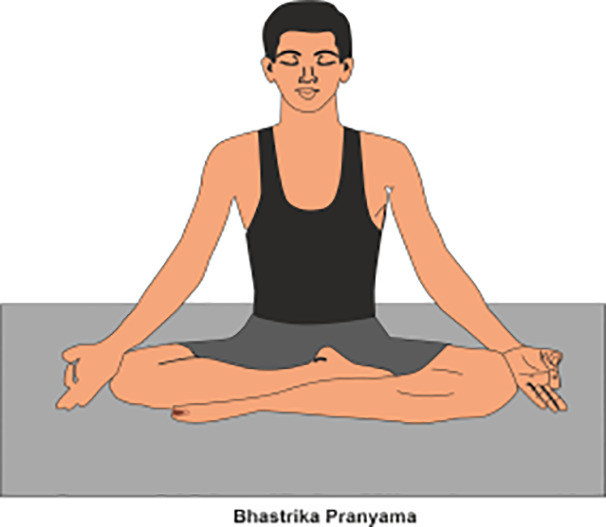
Anulom-Vilom	Also known as alternate nostril breathing, in which practitioner inhaling through one nostril while closing the other, then switching sides during exhalation. This rhythmic practice balances the flow of prana. (Do it 5-10 mins per day)	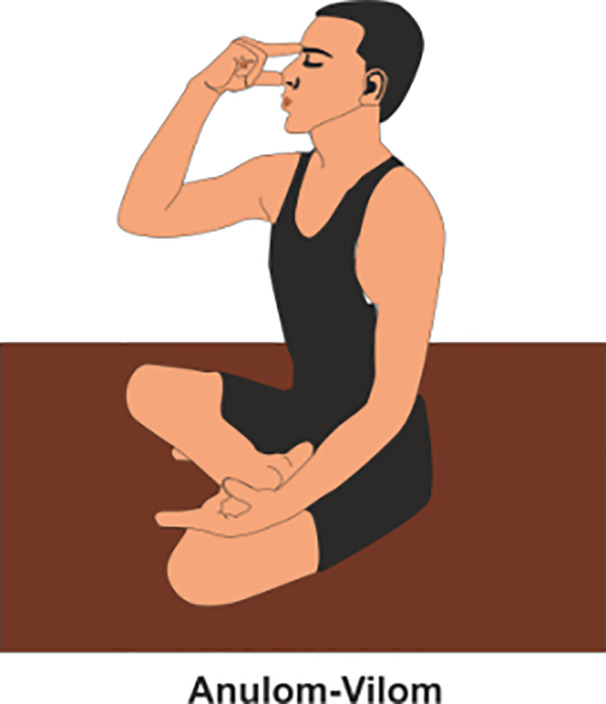
Kapalbhati	Firstly, practitioner Sit comfortably with a straight spine and exhale forcefully through the nose while, pulling the abdomen with each exhale, and allowing the inhalation to happen naturally. (Do it 5-7 mins per day)	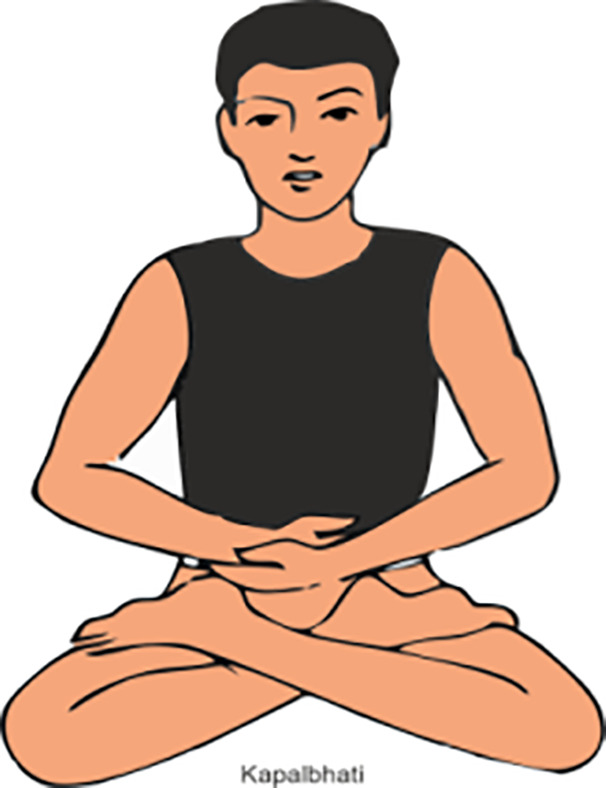

### 6.2 Naturopathy

In the realm of T1DM, naturopathy plays a significant role as a complementary approach to conventional medical treatments. Naturopathic principles emphasize the body’s ability to heal itself, aiming to address the root causes of health issues rather than just treating their symptoms (73). For individuals with T1DM, naturopathic interventions such as dietary modifications, herbal supplements, stress management techniques, and lifestyle adjustments can complement conventional insulin therapy (74) These approaches may help in stabilizing blood sugar levels, improving insulin sensitivity, and reducing the risk of complications associated with diabetes (**Table 3**) (75). Naturopathy can serve as a valuable adjunct to support overall health and well-being in individuals living with T1DM. Naturopathy treatments include mud pack to abdomen, massage to spinal cord and abdomen and cold hip bath (41).

**Table 3:** Different naturopathic therapies which may help in management of Diabetes (75).

Therapies	Method
Mud Application	Make fine paste of soaked mud, applied on the face and body for 20 minutes after which face is washed gently with cold water.
Abdominal and Spinal Massage Therapy	Position your hands on one side of your abdomen. Apply gentle pressure, pushing from one side to the other. Repeat this process for 10-15 min.
Cold Hip bath	Add ice to water until the temperature is between 50°F and 59°F (10°C and 15°C), and allow to patient stay submerged for 10 to 15 minutes
Hot and Cold Compress	Firstly, apply ice for 20 minutes, which cause the vessels to narrow, and then heat for 15 minutes, causing the vessels to dilate. According to patient condition
Sun Bath	Either lie down or sit somewhere in the sun for 15-25 min and relax for a while. Once you start sweating, move from the Sun and then bath in cold water.
Calf Wrap	Wrap each leg “from the feet to the knees” by one wet towel and cover them with the ends of the big towel that lies under the legs. Apply it for 15-20 min.

### 6.3 Ayurveda

According to Ayurvedic texts, all *Pramehas* (urinary disorders including *Madhumeha* or Diabetes mellitus) initiate with the derangement of *Kapha* Dosha that spreads, throughout the body and mixes with *Meda* Dhatu (fat) that is similar in physical properties to *Kapha* Dosha (76). When *Kapha* mixed with *Meda* affects the urinary system, thereby interfering with normal urine excretion. Vitiated other Doshas (e.g. *Vata, Pitta*), and other *Shariric* Dhatu (body tissue) and *Malas* (fluids) may also be involved in this exacerbation of *Kapha* Dosha. Acharya Charaka described the prognosis of the disease *Madhumeha*, described it to be *Kulaja vikara* (meaning a disease occurring due to some genetic defect and hence may be inherited) resulting due to defect in the *Beeja* (either the sperm or the ovum) (77). Sushruta also mentioned the term “*Sahaja*” in context of the genetic predisposition in the pathophysiology of the disease *Madhumeha* (78, 79).

Ayurveda is a traditional system of medicine that originated in India, while its primary focus is on maintaining balance and overall health, some individuals with Type 1 diabetes have explored Ayurveda as a complementary approach to manage their condition. In context of Ayurveda, T1DM diabetes formerly called juvenile diabetes is a chronic condition that is usually diagnosed in children, teenagers and young adults typically require insulin therapy and medical management (80). Maintaining a healthy lifestyle with a balanced diet, regular exercise, and stress management can complement medical treatment for diabetes. Some herbs and supplements that have been studied for potential benefits in managing blood sugar levels include cinnamon, fenugreek, and bitter melon (**Table 4**).

**Table 4:** Some herbs and supplements that have been studied in relation to diabetes management.

Common names	Scientific name	Mechanism	References
Cinnamon	*Cinnamomum verum*	↑ GLUT4 translocation, glucose uptake in insulin-dependent tissues, ↓ phosphoenolpyruvate carboxykinase (PEPCK), glycogen synthase kinase 3β, glucose-6-phosphatase in liver.	(81)
Bitter melon	*Momordica charantia*	↓ levels of Akt, PI3k, TGF-β, JAK2, STAT3, ↑ expressions of PTEN, SOCS3 and SOCS4	(82)
Fenugreek	*Trigonella foenum-graecum*	↑ GLUT4, IR-β, PI3-K	(83)
Aloe vera	*Aloe vera*	↑ B cell receptor, GLUT-4, AMPK, PPARγ	(84)
Madagascar periwinkle	*Catharanthus roseus*	↑ GLUT-4, AMPK, PPARγ	(85)
Berberine	*Berberis aristata*	↑ GLUT-4, AMPK, PPARγ, ↓ PPARα	(86)

## 7 Prevalence of T1DM in India

T1DM is prevalent among pediatric populations, particularly in developing nations such as India, where approximately 97,700 children are affected. Hospital-based studies conducted in India in 1990s indicate that young diabetics, diagnosed before the age of 15, comprise 1%–4% of the total diabetic populace (87). The International Diabetes Federation (IDF) estimates an annual incidence of three new cases of T1DM per 100,000 children aged 0–14 years in India (88). T1DM is experiencing an annual increase of 3–5%. The 10th International Diabetes Federation Atlas of 2021 underscores India’s burden with approximately 2.29 million children aged 0-19 grappling with type 1 diabetes. A subset analysis from the Young Diabetes Registry (YDR) disclosed an average annual incidence rate of 4.9 cases per 100,000 individuals below the age of 20 in Delhi and Chennai (89). Although India has the increasing count of children and adolescents grappling with T1DM, the lack of a comprehensive nationwide registry impedes accurate assessments. Extensive literature research reveals that developing countries face a deficit in structured health initiatives for T1DM, characterized by challenges in implementation and pharmaco-economic viability.

## 8 Discussion

Although T1DM is commonly diagnosed in children and adolescents, it can occur in adults as well, often with a slower onset and milder symptoms (90). T1DM is relatively less prevalent compared to Type 2 diabetes, accounting for about 7–10% of all diabetes cases. Globally, it affects millions of individuals, with a significant number of new diagnoses each year. The prevalence of T1DM varies widely by region, with higher incidences observed in Scandinavian countries, Sardinia, and Kuwait, while lower rates are seen in Asia and Latin America (91). India, despite having a lower prevalence compared to Western nations, still has a notable number of T1DM cases, with approximately one in five children diagnosed with T1DM worldwide being of Indian origin (91). The pathogenesis of T1DM is complex and multifactorial. It is characterized by a progressive decline in insulin levels due to the autoimmune destruction of pancreatic β-cells. This destruction is often triggered by environmental factors in genetically predisposed individuals (92). The presence of specific human leukocyte antigen (HLA) genotypes, such as HLA-DR3-DQ2 and HLA-DR4-DQ8, has been associated with an increased susceptibility to autoimmune β-cell destruction. Other environmental factors, such as viral infections, early exposure to cow’s milk or gluten, and the intake of nitrosamines or nitrates, have also been implicated in the development of T1DM (93). The symptoms of T1DM typically include frequent urination, excessive thirst, unexplained weight loss, and in some cases, diabetic ketoacidosis (DKA), a potentially life-threatening complication. Additionally, T1DM patients often experience significant declines in glycated hemoglobin (HbA1c) levels but less notable decreases in serum cholesterol levels (94). Despite extensive research, no single cause of T1DM has been definitively identified, though ongoing studies continue to explore the various factors that may contribute to its onset. In recent years, there has been a noticeable shift towards integrated therapies for managing T1DM, particularly in India, where traditional healing practices such as Ayurveda, yoga, and naturopathy have long been embraced. These complementary and integrative medicine (CIM) approaches offer holistic pathways to well-being and are increasingly being used alongside conventional treatments to mitigate long-term complications and reduce the costs associated with modern medicine.

Yoga has shown promise in improving the overall health outcomes of individuals with T1DM. Practices such as Asanas (physical poses), Pranayama (breathing exercises), and meditation have been found to positively impact blood glucose control, reduce stress, and enhance insulin sensitivity (15). Similarly, dietary adjustments, regular exercise, and stress reduction techniques are essential components of lifestyle modification that can significantly improve the management of T1DM. For instance, engaging in physical activity has been shown to lower oxidative stress, enhance endothelial function, and improve insulin sensitivity in T1DM patients. Despite the benefits of CIM therapies, it is important to note that they are not intended to replace conventional treatments but rather to complement them (95). The integration of these therapies into diabetes management plans can offer personalized treatment options that address the unique needs and preferences of each patient. However, more research is needed to fully understand the efficacy and mechanisms of these interventions in managing T1DM.

## 9 Conclusion

Type 1 Diabetes Mellitus (T1DM) is a complex, multifaceted disease with widespread effects on individuals and healthcare systems globally. Traditional insulin therapy is essential for managing blood glucose levels and preventing complications; however, it often comes with limitations and challenges, such as variability in patient adherence and long-term risks. In recent years, integrating complementary therapies including dietary modifications, exercise, and mind-body practices like yoga and meditation has shown promise in supporting traditional treatments and enhancing quality of life for individuals with T1DM. Emerging research suggests that these holistic approaches may improve glycemic control, reduce stress, and enhance overall well-being, offering potential benefits that go beyond conventional treatment alone. As the evidence for integrated therapies grows, healthcare providers have the opportunity to empower patients with more comprehensive, personalized management strategies that align with individual lifestyles and preferences. This integrated approach not only aims to optimize clinical outcomes but also fosters patient engagement and resilience, paving the way for a more sustainable and effective management framework for T1DM in the future.

## Data Availability

The original contributions presented in the study are included in the article/supplementary material. Further inquiries can be directed to the corresponding author/s.
